# The Coming of Age of Breast Radiotherapy

**DOI:** 10.3390/curroncol30050392

**Published:** 2023-05-19

**Authors:** Benjamin W. Corn, Shira Galper, Merav Ben-David

**Affiliations:** 1 Faculty of Medicine, Hebrew University, Jerusalem 9103102, Israel; 2 Shaare Zedek Medical Center, Jerusalem 9103102, Israel; 3 Sheba Medical Center, Ramat Gan 52621, Israel; 4 Assuta Medical Center, Tel Aviv 6971028, Israel; meravak@assuta.co.il

Exactly 50 years ago, the investigators of the National Surgical Adjuvant Breast and Bowel Project began to design the B-06 trial [1], a landmark study that compared three treatment alternatives (mastectomy, lumpectomy and lumpectomy with radiotherapy) which were offered to women with relatively early breast cancer. Thus, in 1973, of the architects of that seminal protocol—operating under the audacious leadership of Professor Bernard Fisher—charted a new course for the role of local therapies in the management of breast cancer. In particular, Fisher and his colleagues (predominantly surgeons) boldly deduced that a mutilating operation such as a mastectomy was generally superfluous while conservative surgery (i.e., lumpectomy alone) usually provided insufficient treatment. Oncologists are indebted to these NSABP investigators who interpreted robust data with integrity. The array of articles included in this Special Issue of *Current Oncology* suggest that 2023 represents for a pivotal moment for courageous radiation oncologists.

As editors, we have identified several themes that we deemed to be of interest to the contemporary radiation oncologist.

The concept of “de-escalation” has entered the lexicon of many oncologists [2]. Hence, the long-standing challenge for the radiation oncologist has been the quest to decipher the need for radiotherapy among individual patients after breast-conserving surgery. At a time when various prognostic and predictive gene signatures have enabled more precise treatment recommendations for systemic therapies (chemotherapy as well as endocrine treatments), the parallel development of similar tools should refine the indications for radiotherapy as well. Since radiotherapy comes with the implications of costs and morbidity, such profiling will improve oncologic outcomes including life quality and emotional well-being. In the past [3], poor trial designs mitigated the development of “RT omission classifiers.” Purswani et al. [4] reviewed not only multi-gene profiling but also the classical low-tech parameters (e.g., age and tumor sub-type) considered when attempting to determine the cases for which omission is a reasonable strategy. Meanwhile, Hahn et al. [5] wondered whether or not molecular biomarkers (three were evaluated in their review) could prevent the over-treatment of intraductal carcinoma of the breast. The authors observed that, in contrast to the case of invasive disease, very few protocols have been designed to pursue this type of research with DCIS. Finally, while it is very much customary to contemplate whether or not radiotherapy can be left out of the equation in managing elderly women, McDuff and Blitzblau [6] had the temerity to underscore the toxicities (and non-adherence) associated with endocrine treatment, which prompted them to raise the question of omitting these (morbid and long-course) treatments.

Additional trends were also critically examined by our contributors. For instance, re-irradiation—historically deemed taboo before models emerged to quantify the “temporal dose discount” afforded by the passage of time [7]—was explored by Hardy-Abeloos et al. [8]. The authors offered a primer of different re-irradiation techniques that could be used to treat women who present with either recurrence or a new primary tumor in a previously irradiated breast. Korzets and co-workers [9] looked at internal mammary irradiation, yet another therapeutic approach that was once proscribed by some experts as unacceptable if not improper [10]. By harnessing data from five large prospective randomized trials, they provided a perspective on cardiotoxicity while defining the high-risk patients for whom IMN RT might offer not only the advantage of disease-free survival but also overall survival. One more iconoclastic worldview was presented by Montero and Ciervide [11], who dared to posit the role of *pre-operative* radiotherapy instead of that of the conventional sequence used to integrate the respective modalities prescribed by oncologists.

Two groups contributed to discussions on issues related to oligometastases, a term coined by two luminaries of radiation oncology nearly three decades ago [12]. Franceschini et al. [13] reported mature toxicity data from a phase II trial designed for women with oligometastatic breast cancer manifesting in the liver and lung. They described superb tolerability (e.g., no grade 3 or 4 toxicities in the treatment of 90 lesions among 64 women). In contrast, Freedman et al. [14] elucidated the nuances that distinguish oligometastatic from oligoprogressive stage IV breast cancer. Those authors lamented the reality that despite the rapid advances in technology for stereotactic body radiation therapy, there is still a paucity of level-one evidence attesting to the ability of SBRT to alter the natural history of metastatic breast cancer.

In addition to inviting experts to review and resolve controversial themes for this Special Issue, we also solicited descriptions of innovative topics positioned at the vanguard of breast radiotherapy. Lalani et al. discussed the potential of proton therapy in the management of locally advanced breast cancer [15]. These authors reviewed the unique physical characteristics of proton beams, which may constitute a distinct advantage when clinicians are beset by the need to encompass nodal basins and intricate reconstructions. The report by Maurer et al. [16] was just as pioneering, positing the predictive role of “circulating epithelial tumor cells”. Indeed, using an elegant study design, those investigators observed that patients with an increased CETC/CTC during the course of adjuvant irradiation had inferior disease-free survival in comparison to patients with stable or decreasing CETC/CTC numbers.

The articles assembled for this Special Issue offer a comprehensive overview of the exciting work being carried out by leaders in breast radiotherapy situated around the globe who wish to continue to enhance the current standard of care. Time will be the ultimate judge of precisely which of these thought-provoking ideas will prevail. With the publication of this Special Issue, the readership of *Current Oncology* has the opportunity to review and assess some of these innovative approaches in the radiation treatment of breast cancer.
